# Supplementary material for: Network preservation reveals shared and unique biological processes associated with chronic alcohol abuse in NAc and PFC
Source: PLoS One. 2020 Dec 17;15(12):e0243857. doi: 10.1371/journal.pone.0243857 (PMC7745987; doi:10.1371/journal.pone.0243857)
Supplement: S1 File — (DOCX) [file pone.0243857.s001.docx]

Supplemental methods

**Bi-directional Stepwise Regression:**

The gene expression analysis between our AD cases and matched controls was performed in RStudio (ver. 1.1.463) with the Stats package (ver. 3.5.1) using a bi-directional stepwise regression model for both mRNA and miRNA normalized expression data generated from NAc and PFC. The bi-directional stepwise regression analysis cycles through all available covariates (i.e. age, RIN, pH, PMI, brain weight, hemisphere, toxicology, hepatology, neuropathology status, and smoking) to identify the best-fitting model with the lowest Akaike information criteria (AIC) for each transcript (Mean AIC: NAc= -129.10, PFC= -71.07)

**WGCNA:**

In the WGCNA, our similarity matrix was raised to a power (mRNA β = 14; miRNA β = 6) to approximate the scale-free topography of the adjacency matrix, in which stronger correlations are emphasized over weaker ones. Transcript interconnectedness was determined from the calculated topological overlay measure (TOM). The default, unsupervised hierarchical clustering method was used to partition modules at specified dendrogram branch cut sites using the Dynamic Tree Cut method. Highly correlated modules were then merged based on minimum merge height of r^2^ = .8 and minimum module size of 35. Conventional colors were used to categorically label co-expressed networks and the sum of relative expression within each module is represented as a single value (module eigengene) for downstream phenotypic analysis.

**AUD GWAS Enrichment:**

To compare the overlap between our eQTL results with GWAS of other addiction phenotypes such as for alcohol use and smoking [28, 29]. We began by first isolating all SNPs in LD (r^2=^ 0.50) using the data available from the 1000 Genomes Project [64]. We used Plink 1 [65] to tag SNPs from the 1000 Genomes Project in LD (r2=0.5, -tag-kb=500kb) with significant SNPs from our eQTL analyses (NAc mRNA, NAc miRNA, PFC mRNA, and PFC miRNA). Next, all SNPs from the curated list and those extracted from 1000Genomes were checked against our genotypic dataset to insure that they are all present. The annotations for this expanded list of SNPs was extracted from dbSNP (<https://www.ncbi.nlm.nih.gov/snp/>), which also included SNPs mapped on different dbSNP builds [66]. These steps revealed a total of 10,801 SNPs which were used for the downstream enrichment analysis. This list of SNPs was compared to two different GWAS: i) *Collaborative Study on the Genetics of Alcoholism* (COGA) [28], and ii) *GWAS & Sequencing Consortium of Alcohol and Nicotine Use* (GSCAN) [29]. We performed the Simes Enrichment test [30] on the p-values adjusting for background enrichment between datasets by first, computing the non-centrality parameter of the GWAS Chi-square statistics, then computing the competitively adjusted p-values for each SNP using a Noncentral Chi-square with non-centrality parameter from these adjusted p-values.

**Replication in the GTEx Database:**

We began by performing an independent eQTL analysis on the NAc and PFC from the entire 22,214 probeset and genome-wide SNP data while controlling for all available covariates using the *modelLinear* command within the MatrixEQTL package (ver. 2.2) in R. Next, we isolated significant eQTLs (at FDR ≤0.05) containing SNPs with available RS IDs (NAc=160,119, PFC=35,990) from our sample. This list of eQTLs was then compared to the significant (FDR≤0.05) list of eQTLs in NAc and PFC with available RS IDs and gene symbols (HGNC) (NAc=854,654, PFC=718,679) from GTEx. The overlap significance was tested via a Fisher’s exact test (at p ≤0.05).

**Supplemental Figure 1:** Correlation of module membership (MM) by gene significance within AD associated modules. Grey lines indicate threshold for Hub gene identification MM≥0.80 for AD significant mRNA modules. Each section corresponds with a different brain region and RNA type: A) NAc mRNA, B) PFC mRNA, C) NAc miRNA and D) PFC miRNA.
