# Supplementary material for: Network preservation reveals shared and unique biological processes associated with chronic alcohol abuse in NAc and PFC
Source: PLoS One. 2020 Dec 17;15(12):e0243857. doi: 10.1371/journal.pone.0243857 (PMC7745987; doi:10.1371/journal.pone.0243857)
Supplement: Attachment — Submitted filename: Response to Reviewers_Rev2.docx [file pone.0243857.s012.docx]

**Response to Reviewers (Revision 2)**

**Editor Comments:**Specifically, there is still a concern regarding the low RINs. As one of the reviewers suggests, there could be  more quantitative arguments on this issue. In addition, I could not find the database used for sample access.

*We appreciate the editor’s comments and have uploaded our data to GEO on NCBI. The mRNA and miRNA expression data for the NAc was uploaded as part of a previous study (GSE62699). The PFC mRNA and miRNA data is uploaded and currently being processed, but for the sake of expedience we are resubmitting before obtaining an accession number. We will provide the required accession number once it is released to us.*

**Reviewer #2:** There is lingering concern about the impact of the low RIN samples on the analysis. The authors argue that RIN values are consistently low within PFC (thus impacting cases and controls similarly); however, the concern from the initial review concerned a major goal of the work--to identify overlapping and unique expression differences in PFC and NAc. At a minimum, the proportion of variance explained by the known covariates such as age, gender, RIN and PMI should be reported using violin plots or other graphics. Linear mixed models can then be used to quantify the contribution of multiple sources of variation and identify any covariates that should be corrected in the final analysis.

*We appreciate the reviewer’s comment. As suggested by the reviewer, we have provided a graphical representation (Tables S3) of the contribution of each covariate impact on gene expression. We have also provided further justification on using the bidirectional stepwise regression to correct for the covariates’ effect (Pg. 6 Line 172 & 173) on gene expression (Pg. 9 Line 236-241). Furthermore, while the use of linear mixed models is well developed to adjust for hidden covariates effect in gene associations studies, their applicability in gene expression studies might be less evident (for example see Yao at al. (2019)). We also note that by using stepwise regression, the covariates’ effect is assessed individually on each gene, allowing for the inclusion of only the most relevant covariates, thus minimizing model overfitting. Finally, the validity of our model to correctly capture and adjust for the RIN’s effect on gene expression is reflected by the increased number of times RIN is incorporated as a covariate in the regression models assessing gene expression in PFC vs NAc (See Table S3). Therefore, we are confident in our ability to control for the RIN or any other covariates’ effect in our analyses.*

Yao C, Joehanes R, Johnson AD, Huan T, Liu C, Freedman JE, et al. Dynamic Role of trans Regulation of Gene Expression in Relation to Complex Traits. *Am J Hum Genet* 2017; 100: 571–580.
